# *FOODAlyzer* usability: Advances in food safety education

**DOI:** 10.1371/journal.pone.0333511

**Published:** 2025-10-30

**Authors:** Wen-Li Seow, Kai Wei Lee, Roshariza Haris, Umi Kalsom Md Ariffin, Shing Wei Ng, Sook Yee Lim, Madihah Mohd Saudi, Abdul Rahman Mohamad Gobil, Nurul Azmawati Mohamed, Nor Afiah Mohd Zulkefli, Tengku Zetty Maztura Tengku Jamaluddin, Noris Mohd Norowi, Syafinaz Amin-Nordin

**Affiliations:** 1 Department of Food Service and Management, Faculty of Food Science and Technology, Universiti Putra Malaysia, Serdang, Selangor, Malaysia; 2 Department of Veterinary Preclinical Sciences, Faculty of Veterinary Medicine, Universiti Putra Malaysia, Serdang, Selangor, Malaysia; 3 Department of Medical Microbiology, Faculty of Medicine and Health Sciences, Universiti Putra Malaysia, Serdang, Selangor, Malaysia; 4 Malaysia Research Institute on Ageing, Universiti Putra Malaysia, Serdang, Selangor, Malaysia; 5 CyberSecurity and Systems (CSS) Research Unit, Faculty of Science & Technology, Universiti Sains Islam Malaysia, Bandar Baru Nilai, Nilai, Negeri Sembilan, Malaysia; 6 Center of Computing Sciences, Faculty of Computer and Mathematical Sciences, Universiti Teknologi MARA cawangan Negeri Sembilan Kampus, Seremban, Negeri Sembilan, Malaysia; 7 Microbiology Unit, Faculty of Medicine & Health Sciences, Universiti Sains Islam Malaysia, Nilai, Negeri Sembilan, Malaysia; 8 Department of Community Health, Faculty of Medicine and Health Sciences, Universiti Putra Malaysia, Serdang, Selangor, Malaysia; 9 Department of Multimedia, Faculty of Computer Science and Information Technology, Universiti Putra Malaysia, Serdang, Selangor, Malaysia; 10 Hospital Sultan Abdul Aziz Shah, Universiti Putra Malaysia, Persiaran MARDI-UPM, Serdang, Selangor, Malaysia; University of Lahore - Raiwind Road Campus, The University of Lahore, PAKISTAN

## Abstract

The proliferation of digital technologies offers innovative avenues for public health education. This study evaluates the usability of *FOODAlyzer****©***, a web-based application designed to enhance food safety awareness among Generation Z university students in Peninsular Malaysia. Using a mixed-methods approach, researchers collected data from 419 Gen Z students via online questionnaires. The study comprehensively assessed the app’s effectiveness in improving knowledge, attitudes, and risk perception regarding food poisoning prevention. The results demonstrated significant positive outcomes. Majority of respondents (94.8%) acknowledged *FOODAlyzer©* utility in selecting quality food premises. The app demonstrated strong performance across key usability metrics, with participants reporting high levels of interactivity, accessibility, and ease of learning. Notably, 89.7% of respondents expressed intentions to utilize the app before ordering food, and 90.6% would recommend it to others. While the app demonstrated strong potential, user feedback revealed critical areas for improvement, which were categorized into two main themes: Technical enhancements and food safety education advancements. Key findings highlighted five technical improvement themes. Users called for greater accessibility and inclusivity, including multilingual support, a dedicated mobile app, and features accommodating diverse age groups. Suggestions for interface design focused on improving icon clarity, tooltip implementation, and visual readability. To boost engagement, users recommended an interactive experience with embedded multimedia, peer review systems, and instant feedback mechanisms. Enhancements in functionality, such as a more reliable system and streamlined login process, were also prioritized. Finally, users proposed interconnectedness with external services and government platforms to extend its reach and credibility. Regarding food safety education, three key themes emerge. Users suggested enhancing educational content with clearer criteria to educate both consumers and premise owners. Incorporating reward-based motivation through gamification, quizzes, and promotional incentives was recommended to encourage consistent use. Finally, users emphasized the need for clearer evaluation tools, advocating for simplified question formats, intuitive scales and reduced questionnaire length to minimize user fatigue and improve accuracy. This research provides a user-centered roadmap for developing effective digital public health tools. By addressing these identified limitations and incorporating stakeholder feedback, *FOODAlyzer*© can be optimized to better serve as a model for future digital interventions aimed at improving food safety practices among young adults and the broader community.

## 1. Introduction

The COVID-19 pandemic has dramatically shifted the global attention towards health information, prompting a mass migration of traditional offline events to online platforms. This transition has coincided with a surge in Internet usage, particularly via smartphones [1]. As of January 2023, global smartphone users reached 5.16 billion, representing 64.4% of the world’s population, with 4.76 billion (59.4%) actively using social media. Young people aged 15–24 make up 75% of these users, highlighting the digital native status of younger generation [1].

In Malaysia, smartphone penetration has seen steady growth, increasing from 94.8% in 2021 to 96.8% in January 2023, with projections indicating further expansion in the coming years [2,3]. The country boasts approximately 29 million smartphone users, with younger generations showing higher adoption rates [4]. On average, Malaysian spends 450 minutes daily on the Internet, with 165 minutes dedicated to social media [4]. This digital shift has elevated the importance of smartphone application (app) as effective tools for various purposes, including improving healthy food purchase and enhancing food safety education. Apps have become particularly crucial for reaching technophile generations, acting as modern alternatives to traditional media [4].

However, the adoption of mobile health (m-health) apps in Malaysia faces challenges. A study in Selangor revealed that only 20.4% of M-health app users, particularly millennials, were familiar with the terms and had used health-related apps [5]. While various health apps are commonly accessed for purposes such as fitness tracking, period monitoring and meditation, food safety educational apps were notably absent from popular usage [6].

This gap in food safety app adoption is concerning, especially given the prevalence of foodborne illnesses. Globally, an estimated 600 million cases of foodborne diseases occur annually [7,8]. In Malaysia, 123 food poisoning cases were recorded nationwide in 2021, with unclean food premises identified as a primary cause of outbreak [9].

Recognising the potential of digital tools in public health, Malaysia’s strategic plan for digitalization aims to fully digitize more agency services by 2025. This includes implementing blockchain for streamlining health-related data and upgrading online onboarding programs for offline food and beverage businesses [10]. Given the importance of food safety education and the increasing reliance on digital platforms, there is a clear need for effective, user-friendly apps in this domain. European countries like Denmark, France, Greece and the UK have shown better adoption of food safety education apps, demonstrating their potential impact [11,12].

In light of these developments and challenges, this study aims to evaluate the usability of *FOODAlyzer©*, an integrated technology app designed to advocate food safety education among university students in Peninsular Malaysia. By assessing the effectiveness of this tool, we hope to contribute to the improvement of food safety awareness and practices in the region, ultimately reducing the incidence of foodborne illnesses.

## 2. Methodology

### 2.1 Study design and participants

This study used a mixed-methods approach, combining quantitative and qualitative methods in a cross-sectional design. Prior to data collection, a pilot study was conducted on 4 June 2021 to streamline the data collection process and ensure that the questionnaire was understandable to the general public. A purposive sampling method was used to recruit participants, with data collection taking place from 17 June 2021 to 29 July 2021. The study involved 419 university students at least 19 years old which were born in between mid-1990s and mid-2010s across Peninsular Malaysia, spanning seven states and one federal territory. The inclusion criteria were generation Z Malaysian, currently pursuing tertiary education, using smartphone apps and able to understand the Malay language. Gen Z, also known as post-Millennials, were selected as the study group because they are benchmark of the tech-savvy generation, and they chose to share personal updates through social media [13,14]. University students also frequently dined out, between three to five times per month, and they chose to dine at fast food premises [15]. The exclusion criteria were non-Malaysian and postgraduate students. The sample size for the quantitative component was determined using the Sample Size Calculator (https://www.checkmarket.com/sample-size-calculator/). For the qualitative component, we employed the concept of data saturation to determine the final number of participants for the in-depth interviews.

Next, the current study referred to six main themes in designing the questionnaire. The themes were agreed among authors and were further generated from quantitative answers and investigated deeper through qualitative answers. The six themes included [1] understanding the knowledge, attitude and risk perception of food poisoning prevention, [2] assisting in decision making, [3] ease of use of the app, [4] satisfaction towards the app, [5] subjective quality of the app users and [6] personal innovativeness [16–23]. The pilot test was also carried out to identify the reliability of the questionnaire. The Cronbach’s alpha value among pilot test’s respondents was 0.955 with 72.45 of mean value, which counted as acceptable reliability.

### 2.2 Informed consent and ethical clearance

Written informed consent was obtained before the commencement of the study. Ethical approval was obtained from the Ethics Committee for Research Islamic Sciences University of Malaysia with registered number USIM/JKEP/2019–61.

### 2.3 *FOODAlyzer*© web-based food safety application

**Fig 1:**
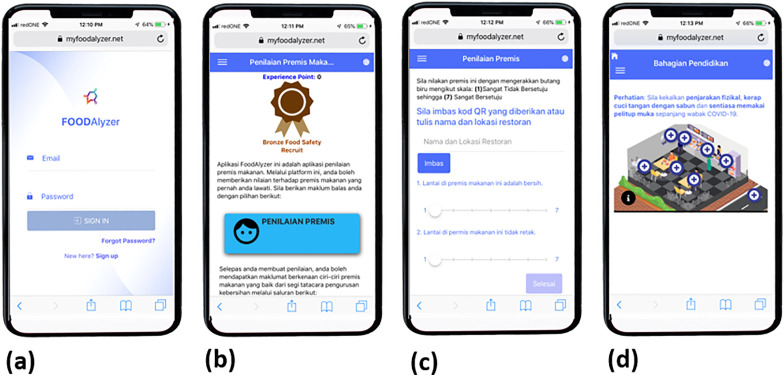
The *FOODAlyzer*© interface employs a layered design: static snapshots provide registration page, introduction and an overview of interactive elements (e.g., + icons).

**Fig 2:**
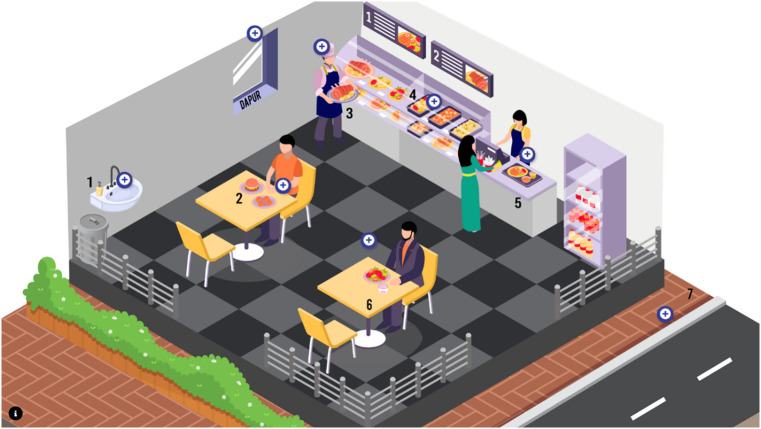
The *FOODAlyzer*© interface employs a layered design: static snapshots provide an overview, while interactive elements (e.g., + icons) link to multimedia explanations (see file:///C:/Users/user/AppData/Local/Microsoft/Windows/INetCache/IE/L59LJMRN/educate[1].html).

*FOODAlyzer©* is an example of web-based food safety application initiated in Malaysia to advocate for consumers’ awareness of the hygiene and sanitation of the food premises by providing online food premises rating and food safety education. Web-based applications are effective in disseminating information and as educational tools in promoting food safety awareness and assisting the food premises selection, thereby reducing foodborne risks [24]. The webpage of the app can be found at https://myfoodalyzer.net/. *FOODAlyzer©* app users have to sign up by using a valid email address and creating a password with no subscription fee. The app has three main sections, [1] the main page, [2] food premises rating by app users and [3] user education about food safety. The main page of *FOODAlyzer©* shows the experience points of the users and a button linked to the food premises rating. To rate the food premises rating, app users can type the name and the address of the food premise or scan the QR code presented at the premise. The rating is based on a seven-point Likert scale with 1 meaning very strongly disagree and 7 meaning very strongly agree. The users should answer all 30 questions on the food premises rating section. After answering the food premises rating, the app users can discover more about the food safety education section. The users can explore a three-dimensional (3D) graphic illustration that animates the environment of a food premise. The 3D visual attention could enable the users to retain the knowledge received and improve understanding of food safety education and app users’ learning achievement [25,26]. Figs 1 and 2 show the screenshot of the web-based app *FOODAlyzer©* and the interface of food safety education section, respectively.

### 2.4 Data collection and questionnaire

Data collection employed a sequential explanatory design which the quantitative data was first collected, followed by qualitative data to provide deeper insights. An online questionnaire was distributed via google forms due to mobile restriction order during COVID-19 pandemic. The questionnaire consisted of 18 Likert-scale questions and one open-ended question, covering six main themes (i.e., 1. Knowledge, attitude, and risk perception of food poisoning prevention; 2. Decision-making assistance; 3. Ease of app use; 4. User satisfaction; 5. Subjective quality; 6. Personal innovativeness) adapted from Okumus et al. [18]. The five-point Likert scale ranged from “strongly disagree” to “strongly agree”, where the positive statements “strongly agree” and “agree” were given “5” and “4” points, respectively. Uncertain answers were marked as “unsure” with “3” points, while the negative statements “disagree” and “strongly disagree” were given “2” and “1” point, respectively.

### 2.5 Data analysis

Quantitative data were analysed for descriptive analysis on IBM Statistical Packages for Social Sciences (SPSS) version 22, and the open-ended data were analysed through Microsoft Excel. Data were presented in n (%) or mean (SD). A p-value less than 0.05 was considered statistics significance.

Qualitative data analysis followed a thematic analysis approach. Two authors independently read through all transcripts to familiarize the contents. Then, researchers generated initial codes from the data that help to develop themes whereby similar codes were grouped into same theme. Followed by themes review and refinement by other researchers and following that clear definitions for each theme were given.

## 3. Results and discussion

### 3.1 Socio-demographic characteristics of *FOODAlyzer*© app users

Table 1 presents the socio-demography characteristics of the *FOODAlyzer©* app users. A total of 419 students participated in the survey, with a notable gender distribution: 72.1% female, and 27.9% male respondents. The majority of participants belonged to Generation Z, a cohort known for being “digital natives; who have grown up with ubiquitous access to technology. Gen Z were more suitable for mobile-learning because they had unlimited access to smartphones and technology gadgets from childhood [14,27]. Regarding academic background, the study sample comprised students from various educational levels. Notably, 80 respondents were enrolled in information technology programs, while the remaining 339 came from diverse fields of study.

**Table 1:** The socio-demographic characteristics of *FOODAlyzer*© app users (n = 419).

Characteristics	Category	N (%)
Gender	Female	302 (72.1)
	Male	117 (27.9)
Study programmes	IT	80 (19.1)
	Non-IT	339 (80.9)
States of institutions of respondents studying	Johor	2 (0.5)
	Kuala Lumpur	19 (4.5)
	Negeri Sembilan	132 (31.5)
	Pahang	1 (0.2)
	Perak	2 (0.5)
	Perlis	6 (1.4)
	Selangor	256 (61.1)
	Terengganu	1 (0.2)

### 3.2 Usability of *FOODAlyzer*©

#### *3.2.1* Understanding of the knowledge, attitude and risk perception of food poisoning prevention.

Majority of the respondents indicated that the *FOODAlyzer*© app effectively enhanced their understanding of food poisoning prevention in terms of knowledge, attitude and risk perception. The statement “*The app helps me understand knowledge regarding food poisoning prevention*” had the highest mean score (mean = 4.66, SD = 0.55), followed closely by understanding of attitude (mean = 4.65, SD = 0.50) and risk perception (mean = 4.61, SD = 0.54) towards food poisoning preventions.

Research suggests that improving food safety knowledge positively influences users’ attitude towards food poisoning prevention [28]. Furthermore, the heightened risk perception of food poisoning preventions positively affected with a more positive attitude toward food poisoning prevention measures [29]. To align with Sustainable Development Goal (SDG) 3, which aims to ensure healthy lives and promote well-bring, intervention using 3D visualisation tool have shown promise in enhancing app users’ food safety knowledge and attitudes towards food poisoning prevention [30].

The COVID-19 pandemic has amplified concerns about food-related infection risks, as the public initially feared that the virus could be transmitted through food [31]. To address the knowledge-behaviour gap in safe food handling, studies have found that interactive online games are more effective than video-based activities. These online games provide an engaging environment for users to repeatedly practice correct behaviour [32].

#### 3.2.2 Assisting in decision making.

The study results indicated that the app proved advantageous in helping users select quality food premises for dining out or taking away (mean = 4.54, SD = 0.60). *FOODAlyzer©* app serves as a tracking tool, allowing users to scan QR codes provided by each premise and evaluate its the cleanliness of the premise through food premises rating. The overall cleanliness ratings provided by the app users could be utilised as a tracking tool for diners and local authorities in choosing and monitoring cleanliness of the food premises, respectively [33].

Consumers preference leans towards customised app information that aligns with their values and needs. Key factors influencing the use of digital food apps include clean premises, proper food hygiene, food safety, food freshness, and overall food quality [34]. The advent of industry 4.0 technologies, including artificial intelligence (AI), Internet of Things (IoT), robotics, real-time data and big data have been boosting food safety and promoting unique food education campaigns [35,36]. For example, AI-powered apps can assist users to decide better when choosing quality food premises by monitoring food quality, temperature and cleanliness, and ensuring the food handlers adherence to personal protective equipment protocol [36].

#### 3.2.3 Ease of use of the app.

The ease of use of the *FOODAlyzer©* app was evaluated based on three sub-subjects: learnability, interactiveness and accessibility. The learnability sub-subject had the highest score in which the statement of “*The app is easy to learn*” had the highest marks (mean = 4.45, SD = 0.69). In term of interactiveness, the statement of “*The interaction and navigation through the app is straightforward (intuitive)*” (mean = 4.25, SD = 0.82) had a lower total mean score, indicating room for improvement in this area.Followed by “*Instructions in the app is easy to follow*” (mean = 4.36, SD = 0.75). These results suggest that *FOODAlyzer©* provides an accessible approach particularly for younger users. But accessibility (mean = 4.24, SD = 0.80) of *FOODAlyzer©* was the least preferred aspects among respondents with some users reporting unpleasant experiences such as repeated login requirement and refresh issues.

Research indicates that the quality of a website, download speed, and ease of navigation play crucial roles in fostering an image of clean and hygienic food premises [37]. Young adults (18–25 years) preferred to use applications that are reliable, simple and objective [22]. In the food service sector, app user prioritize functions that are easy to use and learn, consistency and simple to understand [34]. Furthermore, user-friendly apps with improved performance positivity influence users’ intentions to continue using and adopting the apps [18,38,39]. Moreover, easily usable apps can enhance learning performance and satisfaction in mobile learning contexts [38].

#### 3.2.4 Satisfaction towards the app.

Improving user satisfaction towards *FOODAlyzer©* requires enhancing the apps’ loading speed. The statement “*The loading speed of the app is fast*” (mean = 4.17, SD = 0.87), with an increased number of respondents disagreeing with this assertion. Users reported issues such as inability to refresh the web page, had blank page when logged in, or difficulties logging into the web-based app. These issues likely due to the huge volume of concurrent connection during peak usage periods, affecting apps’ performance [17]. Internet connectivity is also important for maintaining loading speed as weak connection and adverse weather conditions can impact performance [40]. Hence, key factors influencing user satisfaction include information quality, service quality and system quality [37]. Apps that frequently lag, hang or have slow loading speeds between pages can significantly reduce user satisfaction [41].

#### 3.2.5 Subjective quality of the app users.

Subjective quality refers to the app users’ perspectives on the app [42,43]. *FOODAlyzer©* app users provided positive feedback regarding this subject. In term of two statements for app engagement, the statement “*The app is interesting for repeated use*” had the highest mean mark (mean = 4.29, SD = 0.71), while the statement “**Given the chance, I intend to use* FOODAlyzer© *App before ordering food and beverages in restaurants**” recorded the lowest mean mark (mean = 4.25, SD = 0.68) with 34 respondents (8.1%) choosing “3-Unsure” for this statement. Thus, these results indicated that *FOODAlyzer©* meets user expectations and motivates them to continue using the apps in the future [37]. Positive and negative dining experiences—such as poor food quality, sanitation and food safety issues—can encourage individuals to voice their concerns. Therefore, by offering a dedicated platform to raise awareness about food safety, *FOODAlyzer©* can motivate users to remain engaged with the app. The web-based app users’ intention to revisit the apps increased when they felt pleasure and interest, and they could control the specifications of the apps [44]. Engaging apps can also improve app users’ learning performances [38]. Interestingly, while most respondents found *FOODAlyzer©* app interesting for revisiting, fewer indicated they would practically use it before ordering food. This discrepancy suggests that emotional attachment to the app may be stronger than its practical application.

Users showed a willingness to introduce (mean = 4.26, SD = 0.70) and recommend the app to others (mean = 4.28, SD = 0.69) for general use and (mean = 4.28, SD = 0.61) for restaurant use. These findings highlights the importance of social influence in app adoption and use [45]. Well-designed apps can effectively connect users with trusted individuals in terms of intelligence, awareness, and educational level [18].

#### 3.2.6 Personal innovativeness.

For the respondents’ personal innovativeness in driving them to use *FOODAlyzer©*, three statements, “*I like trying new app*” (mean = 4.07, SD = 0.94); “*If I heard about newly-introduced app, I would try using it*” (mean = 3.98, SD = 0.91) and “*I would always be the first to try newly introduced app among my peers*” (mean = 3.76, SD = 1.03) scored the lowest marks.

These results showed that while respondents generally enjoy trying a new apps, they are not necessarily early adopters. This finding may be attributed to a preference for more fun, joyful and enjoyable apps [46]. Continuous app use is influenced by user satisfaction, trust, and the absence of space and time restrictions [38]. The personal habits and frequency of app use positively impact the intention to continue using the apps during and even after the pandemic [47]. Thus, the stronger the intention of try new apps, the greater the likelihood of actual adoption [48].

### 3.3 Overall rating

The respondents were asked to rate *FOODAlyzer©* using experience. “*Rate your experience using the app*” (mean = 4.37, SD = 0.71) earned positive response from the *FOODAlyzer©* respondents. For further improvements, the respondents were invited to evaluate their suggestions and comments on *FOODAlyzer©*’s** improvements. Table 2 shows the quantitative data of *FOODAlyzer© usability.*

**Table 2:** Usability of *FoodAlyzer*©*.*

Main Themes	Measurement Items	Sample size, *N* = 419 (%)					Mean	SD
**Strongly disagree**	**Disagree**	**Unsure**	**Agree**	**Strongly agree**		
Understanding in knowledge, attitude and risk perception of food poisoning prevention	1. The app helps me understand knowledge regarding food poisoning prevention	2 (0.5)	0 (0)	4 (1.0)	128 (30.5)	285 (68.0)	4.66	0.55
	2. The app helps me understand attitude regarding food poisoning prevention	0 (0)	1 (0.2)	1 (0.2)	141 (33.7)	276 (65.9)	4.65	0.50
	3. The app helps me understand risk perceptions regarding food poisoning prevention	1 (0.2)	0 (0)	6 (1.4)	148 (35.6)	264 (63.0)	4.61	0.54
Assisting in decision making	4. The app can be advantageous in choosing quality food premises	0 (0)	2 (0.5)	17 (4.1)	154 (36.8)	246 (58.7)	4.54	0.60
Ease of use of the app (learnability, interactiveness and accessibility)	5. The app is easy to access	3 (0.7)	12 (2.9)	41 (9.8)	189 (45.1)	174 (41.5)	4.24	0.80
	6. The app is easy to learn	4 (1.0)	4 (1.0)	11 (2.6)	181 (43.2)	219 (52.3)	4.45	0.69
	7. The interaction and navigation through the app is straightforward	3 (0.7)	15 (3.6)	37 (8.8)	183 (43.7)	181 (43.2)	4.25	0.82
	8. Instructions in the app is easy to follow	1 (0.2)	14 (3.3)	20 (4.8)	183 (43.7)	201 (48.0)	4.36	0.75
Satisfaction towards the app	9. The loading speed of the app is fast	3 (0.7)	19 (4.5)	53 (12.6)	172 (41.1)	172 (41.1)	4.17	0.87
Subjective quality of the app users	10. I will introduce this app to others	2 (0.5)	2 (0.5)	43 (10.3)	212 (50.6)	160 (38.2)	4.26	0.70
	11. I will recommend others to use this app	2 (0.5)	2 (0.5)	38 (9.1)	210 (50.1)	167 (39.9)	4.28	0.69
	12. Given the chance, I intend to use *FoodAlyzer* App before ordering food and beverages in restaurants	1 (0.2)	6 (1.4)	34 (8.1)	224 (53.5)	154 (36.8)	4.25	0.68
	13. Given the chance, I plan to suggest this app to other people when ordering food and beverages in restaurants	1 (0.2)	3 (0.7)	33 (7.9)	224 (53.5)	158 (37.7)	4.28	0.66
	14. The app is interesting for repeated use	1 (0.2)	6 (1.4)	40 (9.5)	197 (47.0)	175 (41.8)	4.29	0.71
Personal innovativeness	15. I like trying new app	7 (1.7)	26 (6.2)	50 (11.9)	184 (43.9)	152 (36.3)	4.07	0.94
	16. If I heard about newly-introduced app, I would try using it	5 (1.2)	27 (6.4)	70 (16.7)	190 (45.3)	127 (30.3)	3.98	0.91
	17. I would always be the first to try newly introduced app among my peers	10 (2.4)	47 (11.2)	87 (20.8)	168 (40.1)	107 (25.5)	3.76	1.03
Overall	18. Rate your experience using the app	0 (0)	8 (1.9)	34 (8.1)	185 (44.2)	195 (45.8)	4.34	0.71

### 3.4 Improvements on *FOODAlyzer*©

The respondents’ suggestions were summarised into two main categories of eight themes, [1] technical improvements suggested for *FOODAlyzer©* web-based app (five themes), and [2] food safety education and food premises rating improvements (three themes).

#### 3.4.1 Technical improvements suggested for *FOODAlyzer*© web-based app.

*3.4.1.1 Accessibility and inclusivity of use.* Four respondents highlighted the user friendliness across different demographics, such as need for larger fonts, more intuitive icons, and simplified navigation to accommodate users of all ages, particularly the elderly. Furthermore, multilingual access was mentioned by the respondents (*n* = 4) through adding more language options. The respondents emphasized the importance of inclusivity by incorporating more languages (e.g.,: Malay, Mandarin, Tamil) to reach diverse user groups. Many respondents (*n* = 18) also suggested device and app platform compatibility improvement in which providing downloadable standalone app. This may due to Gen Z respondents preferred a mobile app downloadable via the App Store instead of web-based browser access, enhancing convenience and accessibility.

“**This application may not be suitable to people who are 50 years old and above, because this group of people needs easier and briefer way to access the apps. For example, they may face issue if they want to access YouTube from* FOODAlyzer©*. Perhaps, old people do not familiar with the apps setting and they are not clear on how to click back to the main page.**” (Respondent 179)

“*The apps should be bilanguage or given a selection of language.*” (Respondent 158)

“*Mobile app version from App Store/Play Store is better compared to web-based app. As it is easier to use and access.*” (Respondent 105)

Despite more than half of the senior citizens (61.9%) hesitant about internet use due to perceived technological limitations, 81.1% still express interest in receiving health related information through online platform [49]. *FOODAlyzer©* should also provide multilingual support to accommodate Malaysia’s diverse population, improving accessibility across diverse users’ backgrounds, including different age groups, mother tongues, preferred languages and educational backgrounds. Almost every Malaysian (96.8%) has access to the Internet, and 86.3% download software and apps [49], making *FOODAlyzer©* available on Apple App Store and Google Play Store would increase user acquisition. These suggest a significant opportunity to design an accessible and inclusive app that bridges technological gaps.

*3.4.1.2 Interface design and visual communication.* Four respondents had similar concern in app icon clarity and consistency due to confusing ‘+’ icons in *Pendidikan* section. The participants found that similar-looking icons created confusion while using the app, suggesting the need for unique and clearly labelled icons. In addition, enhancement in visual readability were suggested (*n* = 5) due to small infographic posters and small font size. Infographics were too small to read on smartphones, undermining food safety educational purposes of the content.

“*This app is very good and I would like to suggest that each button that can be clicked on the app should provide a tooltip, a brief info of the button. Thus, it will be easier for the app users to understand.*” (Respondent 128)

“*Quite confuse when going back to main hall from kitchen because the sign + is the same, maybe can change to others for better experience. The info graphic posters are very small to be read on smartphones, maybe can enlarge it or do some interactive way to educate people.*” (Respondent 6)

Thus, clear icons and tooltip guides are essential to help app users understand food safety criteria and accurately rate premise cleanliness. Food safety educational materials, such as posters should be easily navigated through intuitive icons and in lager font size.

*3.4.1.3 Interactive and engaging user experience.* The respondents (*n* = 4) were looking for engaging virtual environment, suggesting feedback and review mechanisms on *FOODAlyzer©* that equipped with comment section and peer reviews. Accessing to other users’ evaluation and providing personal feedback on food premises’ hygiene and sanitation level may enhance transparency evaluation. Secondly, a functional multimedia integration could be integrated by embedded YouTube viewing on *FOODAlyzer©* app. The respondents (*n* = 3) commented the issues being redirected to YouTube, interrupting the experience, and hence suggested to embed video playback to retain engagement. To further, one respondent (Respondent 137) suggested actionable feedback for app users by popping up the summary of evaluation results, requesting an instant feedback post-evaluation, such as whether the food premise is suitable for dining, which enhances the engagement between the app and users.

“*My suggestion is to list all the restaurants and have a search button to make it easier for users to find and see the cleanliness analysis of a restaurant. You can also add a comment and rating section so that users can more easily look at user satisfaction with the restaurant.*” (Respondent 36)

“*No need to switch to YouTube to watch the videos, can just use the current app.*” (Respondent 7)

“*After the user evaluates, the system can inform whether the user is suitable to have meals at the evaluated restaurant based on their evaluation.*” (Respondent 137)

Multiple functions and advanced features can attract higher user volume and meet the needs of the users. These apps should integrate purposes of app ranging from creating awareness to offering notice cautions and managing food poisoning issues, unlike current common featured app. The app users were able to engage in food safety educational information when exposed to both cognitive and affective learning structures [50]. Hence, an evidence-based approach is critical to upholding the accuracy of the food safety information conveyed through expert inputs, which boosts *FOODAlyzer©* as a trusted pioneer app for the users to decide and consume from safe and hygiene food premises [43].

*3.4.1.4 Functionality and system performance.* System responsiveness and reliability due to app frozen or logged out automatically were highlighted by the respondents (*n* = 3). When navigating certain features like YouTube videos, users experienced being logged out, indicating app instability that can disrupt the performance of *FOODAlyzer©*. In addition, the respondents (*n* = 7) suggested to improve login and evaluation flow by enabling phone number login and faster email verification. A smoother login experience was recommended to increase system performance and to reduce user frustration.

“*This app keeps log me out after I click on the YouTube video.*” (Respondent 165)

“*Change the sign in way from email switch to phone number so it’s easier to log in.*” (Respondent 40)

Time-saving while using the apps was the primary factor that affected the app users to keep using the app because time-saving apps could increase the performance of the app and the tendency of the app users to browse the apps [47]. Nevertheless, majority of the app users preferred apps that could respond effectively and focus on user-centred design [43].

*3.4.1.5 Interconnectedness with broader food and food safety related ecosystem*. One respondent urged for external service integration by linking with “*foodpanda”* or other delivery platforms. Connecting with food delivery apps could extend the reach of food safety messages in a larger volume of app users. Moreover, alignment with government existing infrastructure (*n* = 2), such as *MySejahtera* app which could enhance credibility and adoption of *FOODAlyzer©*.

“*Link *with* “foodpanda” and other food-delivery services.*” (Respondent 76)

“*Conduct two-way collaboration with the Malaysian government through the Ministry of Communications to improve this application and promote extensively like MySejahtera application*.” (Respondent 88)

Collaboration with local-focused government authorities and science institutions would enhance users’ trust and credibility towards the app [46]. An evidence-based approach is also critical to upholding the accuracy of the food safety information conveyed through expert inputs, which boosts *FOODAlyzer©* as a trusted pioneer app for the users to decide and consume from safe and hygiene food premises [38].

#### 3.4.2 Food safety education and food premises rating improvements.

*3.4.2.1 Enhancing food safety education*. Two respondents suggested to initiate targeted educational features for the criteria for clean food premises in ‘*Pendidikan*’ section by adding important food safety cleanliness criteria to educate both consumers and premise owners, reinforcing behaviour change towards better hygiene practices.

“*In my opinion, it may be necessary to add a few more detailed criteria regarding what needs to be in a restaurant to make it of better quality in terms of restaurant cleanliness.*” (Respondent 121)

“*Hopefully this application will also provide information to food premise owners about the practices that should be taken to maintain food hygiene and cleanliness of the premises.*” (Respondent 138)

Internal and external hygiene factors, such as food handlers’ socio-demographic characteristics, knowledge, attitude, and food handling premises could affect hygiene practices of the foodservice premises [51]. Furthermore, poor food safety knowledge level among migrant food handlers in Malaysia highlighted the importance of advocating food safety education among respective group of population [52]. On top of that food safety criteria were not prioritised by consumers when selecting food premises although food safety information is important as a guideline during selecting premises to dine [24].

*3.4.2.2 Reward-based user motivation.* Gamification and incentives, such as offering interactive quizzes and points on *FOODAlyzer©* (*n* = 3) could enhance food safety educational purposes. The respondents (*n* = 2) also expected promotional benefits like coupons or rebates for frequent raters of the app. This is due to incentivizing evaluations through tangible rewards could encourage repeated app use and consistent feedback contributions.

“*Simple quiz and games can be integrated into the app to test users’ understanding of food poisoning prevention subjects.*” (Respondent 28).

“*Give promotion coupon or money rebate to the person who often rates the restaurant*.” (Respondent 50)

Online game-based activity could enhance consumers food safety behaviours and beliefs in which game showed less impact on beliefs among consumers ages 20–30, however, surprisingly it showed better impact for the older age groups [32]. Furthermore, implementation of tablet PC simulation game proved that college students were motivated in learning food safety knowledge [50].

*3.4.2.3 Clarity and effectiveness of evaluation tools.* One respondent pointed a critical issue of confusion during evaluation intent, ambiguity between self-evaluation and premise evaluation. The respondents were unsure whether they were rating their own perceptions or the premises themselves, signalling a need for clearer instructions for the ‘*Penilaian Permis*’ questionnaire. Secondly, improvement of questionnaire scale format should be considered by replacing Likert scale with emoticons or descriptive options. Gen Z respondents (*n* = 10) suggested more intuitive, simplified formats, such as emojis and Yes/No that are easier to interpret and less prone to error. Thirdly, length and complexity of questionnaire with too many and long questions in the questionnaire (*n* = 11) should be improved to reduce user fatigue, for ease of understanding and to improve completion rates.

“*The questions for “Penilaian” section are confusing. The rate is for the shop or for our perspective*?” (Respondent 5)

“*Instead of scale, maybe can use emoticon icon to show the reaction when evaluate the restaurant*.” (Respondent 67)

“*Do not use a long sentence for the evaluation part. Using just keyword is better because people usually don’t like to read long sentences*.” (Respondent 43)

Although online and offline interventions showed no difference in improving respondents’ food safety and handling knowledge, however the knowledge scores of the respondents declined with increasing in intervention number. This shows that identifying optimum intensity while answering the questionnaire on *FoodAlyzer*©** could reduce the tendency of user fatigue [53]. Combination of diverse learning and evaluation strategies, such as surveying, case-based learning, simulation-based learning, gamification and situated learning could also positively impact knowledge, attitude and behavioural changes of the participants involved [54] (Table 3).

**Table 3:** Summary of improvement’s themes and sub-themes descriptions.

Category	Theme	Sub-Theme	Quote
Technical improvements	Accessibility and inclusivity of use	User-friendliness across demographics	“*This application may not be suitable to people who are 50 years old and above, because this group of people needs easier and briefer way to access the apps. For example, they may face issue if they want to access YouTube from FoodAlyzer*©*. Perhaps, old people do not familiar with the apps setting and they are not clear on how to click back to the main page.*” (Respondent 179)
		Multilingual access	“*The apps should be bilanguage or given a selection of language.*” (Respondent 158)
		Device and platform compatibility	“*Mobile app version from App Store/Play Store is better compared to web-based app. As it is easier to use and access.*” (Respondent 105)
	Interface design and visual communication	Icon clarity and visual consistency	“*This app is very good and I would like to suggest that each button that can be clicked on the app should provide a tooltip, a brief info of the button. Thus, it will be easier for the app users to understand.*” (Respondent 128)
		Visual readability	“*Quite confuse when going back to main hall from kitchen because the sign + is the same, maybe can change to others for better experience. The info graphic posters are very small to be read on smartphones, maybe can enlarge it or do some interactive way to educate people.*” (Respondent 6)
	Interactive and engaging user experience	Feedback and review mechanisms	“*My suggestion is to list all the restaurants and have a search button to make it easier for users to find and see the cleanliness analysis of a restaurant. You can also add a comment and rating section so that users can more easily look at user satisfaction with the restaurant.*” (Respondent 36)
		Functional multimedia integration	“*No need to switch to YouTube to watch the videos, can just use the current app.*” (Respondent 7)
		Actionable feedback for app users	“*After the user evaluates, the system can inform whether the user is suitable to have meals at the evaluated restaurant based on their evaluation.*” (Respondent 137)
	Functionality and system performance	System responsiveness and reliability	“*This app keeps log me out after I click on the YouTube video.*” (Respondent 165)
		Improve login and evaluation flow	“*Change the sign in way from email switch to phone number so it’s easier to log in.*” (Respondent 40)
	Interconnectedness with broader food and food safety related ecosystem	External service integration	“*Link with “foodpanda” and other food-delivery services*.” (Respondent 76)
		Alignment with government existing infrastructure	“*Conduct two-way collaboration with the Malaysian government through the Ministry of Communications etc. to improve this application and promote extensively like MySejahtera application*.” (Respondent 88)
Food safety education and food premises rating improvements	Enhancing food safety education	Targeted educational features	“*In my opinion, it may be necessary to add a few more detailed criteria regarding what needs to be in a restaurant to make it of better quality in terms of restaurant cleanliness.*” (Respondent 121)
			“*Hopefully this application will also provide information to food premise owners about the practices that should be taken to maintain food hygiene and cleanliness of the premises.*” (Respondent 138)
	Reward-based user motivation	Gamification and incentives	“*Simple quiz and games can be integrated into the app to test users’ understanding of food poisoning prevention subjects.*” (Respondent 28)
		Promotional benefits	“*Give promotion coupon or money rebate to the person who often rates the restaurant*.” (Respondent 50)
	Clarity and effectiveness of evaluation tools	Confusion during evaluation intent	“*The questions for “Penilaian” section are confusing. The rate is for the shop or for our perspective*?” (Respondent 5)
		Improvement of questionnaire scale format	“*Instead of scale, maybe can use emoticon icon to show the reaction when evaluate the restaurant*.” (Respondent 67)
		Length and complexity of questionnaire	“*Do not use a long sentence for the evaluation part. Using just keyword is better because people usually don’t like to read long sentences*.” (Respondent 43)

## 4. Limitations and suggestions for future research

While this study provides valuable insights into *FOODAlyzer©*’s usability, several limitations should be noted. First, the recruitment of participants via digital channels (e.g., online surveys, app-based testing) may have introduced selection bias, as only individuals comfortable with internet and app usage could participate. This limits the generalizability of findings to non-tech-savvy populations, such as older adults or those with limited digital literacy. However, this approach was intentional to align with the study’s objective of evaluating the app among its primary target users (tertiary students and younger demographics). Future research could employ mixed-method recruitment (e.g., community outreach) to include broader populations. Additionally, the use of purposive sampling (focusing on Malay-speaking tertiary students) ensured internal validity for assessing usability within this key demographic but may not reflect the experiences of other user groups. We recommend follow-up studies with randomized sampling across diverse age, education, and socioeconomic backgrounds to strengthen external validity.

Apart from the main findings, the study acknowledges potential areas for future development of *FOODAlyzer©*. The ease of use and learnability of sub-subjects should be improved particularly for commercialization. In addition, timing logging function can be considered to implemented in the app to measure the duration of app users to complete the food premises rating. Special attention should be given to advanced age groups who prefer simple instruction and user-friendly interfaces. Data privacy is another significant concern, with the current app design lacking comprehensive privacy features or polices that may rise users concerns. To ensure compliance with data protection regulations, Malaysia app developers must strictly adhere to the PDPA 2010 requirements while also considering GDPR provisions for potential global expansion. This involves implementing robust privacy measures including data minimization practices that collect only essential user information, as well as establishing clear breach notification protocols [55]. From a technical perspective, publishing *FOODAlyzer©* on major app distribution platforms like Google Play store and IOS App Store would significantly improve accessibility and adoption rates. Concurrently, optimizing the application’s memory footprint would enhance its compatibility across diverse smartphone devices with varying storage capacities. Future studies could explore integrating gamification elements and AI-driven solution into the app to enhance food safety education for digital users. These technologies show particular promise for enhancing food safety education, improving traceability across supply chains, and strengthening fraud detection capabilities [56]. Furthermore, gamification was proven effective in engaging students through simulated foodborne outbreak scenarios while enhancing their problem-solving skills [57]. Additionally, incorporating a directory of official government contacts would enable users to promptly report suspected food poisoning cases or outbreaks to relevant authorities.

## 5. Conclusion and take-home message

While *FOODAlyzer©* has room for improvement, the current study represents a significant step towards addressing inadequate food safety education among Malaysians. Web-based apps similar to *FOODAlyzer©* demonstrate considerable potential for effectively advocating food safety education in the long term. *FOODAlyzer©* is not intended to be a standalone solution but rather a collaborative tool. The potential for partnerships with various authorities offers a two-way improvement strategy. The positive feedback received from the respondents suggests that *FOODAlyzer©* can effectively promote food safety education in the food service industry through a digitalised medium.
